# An oral combined contraceptive user with elevated D-dimer post COVID-19: a case report

**DOI:** 10.1186/s12905-021-01456-5

**Published:** 2021-08-28

**Authors:** Nada A. Alyousefi

**Affiliations:** 1 Department of Family and Community Medicine, College of Medicine, King Saud University, Riyadh, Saudi Arabia; 2 King Saud University Medical City, King Saud University, Riyadh, Saudi Arabia

**Keywords:** Contraception, COVID-19, Deep venous thrombosis, OCP, Venous thromboembolism, Case report

## Abstract

**Background:**

This case discusses the challenges created by COVID-19 (coronavirus disease 2019) in the area of hormonal contraception, highlighting the contraception knowledge gap for women in their post COVID-19 period, especially if they had high D-dimer levels.

**Case presentation:**

This case involves a thirty-eight-year-old woman taking combined oral contraception (desogestrel/ethinyl oestradiol tablets) with a history of varicose veins. She recovered from a COVID-19 infection in November 2020. She presented to the emergency room with right lower-limb pain below the knee and progressive swelling for five days in February 2021. Physical examination of the lower limb showed mild swelling and tenderness of the right leg compared to the left leg. D-Dimer was elevated (1.06 mcg/mL FEU). COVID-19 screening was negative. A Doppler scan to exclude DVT was performed considering the clinical picture and high D-dimer level. There was no evidence of DVT in the right limb. She was reassured and discharged with instructions on when to visit the emergency room. The D-dimer had decreased to 0.53 mcg/mL FEU in March 2021. She booked an appointment with family medicine clinics because she was concerned about the continuation of combined oral contraception (desogestrel/ethinyl oestradiol tablets) with high D-dimer and risk of thrombosis. The follow-up D-dimer level in May 2021 was normal (0.4 mcg/mL FEU). The patient preferred to continue taking oral contraception.

**Conclusion:**

An evidence-based consensus is needed to guide clinicians in providing contraception counselling for such patients.

## Background

Coagulopathy is common in patients with severe COVID-19, though the mechanisms are not fully understood [1–3], and the significance of persistent elevation in D-dimer in some recovered COVID-19 patients remains unknown. All combined contraceptives analysed in a Cochrane review were associated with an increased risk of venous thromboembolism [4]. However, it is unclear whether hormonal contraception use among COVID-19-positive women increases the risk of thromboembolism [5]. This case report describes how challenging it is to provide contraception counselling for post-COVID-19 patients, especially those with elevated D-dimer.

## Case presentation

The patient was a thirty-eight-year-old woman and mother of three children who delivered her youngest child in May 2019. She resumed her combined oral contraception in November 2019. She had a history of controlled bronchial asthma and hypothyroidism and used a budesonide-formoterol inhaler and levothyroxine tablets.

She also had a history of varicose veins. In January 2020, a venous duplex scan showed a right long saphenous vein incompetent from the above knee. There was no evidence of deep vein thrombosis (DVT) or deep vein reflux. She was treated by a vascular surgeon in ambulatory care clinics in February 2020, with a plan for an elastic stocking and injection sclerotherapy. Ensuring follow-up appointments were cancelled because of the COVID-19 pandemic.

In November 2020, she presented to the emergency room due to three days of tiredness, aches and pains, and loss of taste and smell. Her swab test for COVID-19 was positive. Home isolation with paracetamol and albuterol inhaler puffs for mild shortness of breath were given. No hospitalization was required. The shortness of breath symptoms resolved within days, and her senses of taste and smell recovered after 1 month.

In February 2021, she presented to the emergency room with a history of right lower-limb pain below the knee and progressive swelling for five days. The pain had increased in severity for one day; she took paracetamol, but the pain did not improve. There was no history of fever, shortness of breath or chest pain, no recent trauma or surgery, stroke, DVT, cancer, skin change, prolonged immobility, or pregnancy; she had not travelled recently. She had been taking regular oral combined contraception (OCP), i.e., desogestrel/ethinyl oestradiol tablets, since resuming in November 2019. There was no history of contact with a confirmed case of COVID-19.

Physical examination showed a temperature of 36.5 °C (oral), respiratory rate of 19, blood pressure of 121/79, SpO2 of 99%, weight of 59 kg and BMI of 23.63. The patient was conscious, alert, and oriented. Cardiovascular exam was normal, as were chest and abdomen examinations. The lower limb of the right leg showed mild swelling and tenderness in comparison to the left leg. Her neurovascular examination was normal. Limb measurements were as follows: right calf muscle 36 cm; right thigh muscle 49; left calf muscle 35; and left thigh muscle 48. There was a normal range of motion; she had mild swelling in the right lower limb with no erythema.

COVID-19 screening, which was performed per the emergency room admission protocol, was negative. According to laboratory findings, WBC, RBC, Hgb, Hct, MCV, MCH, MCHC, RDW, platelet, MPV, renal profile, liver, and thyroid function tests were all within normal limits. However, D-dimer was high at 1.06 mcg/mL FEU (the normal range is 0.22–0.45).

A Doppler scan to exclude DVT was performed considering the clinical picture and high D-dimer level. There was no evidence of DVT in the right limb vein. She was reassured and discharged with instructions to visit the emergency room. A follow-up appointment with vascular surgery was scheduled in 1 week.

When presenting to vascular surgery follow-up appointments, the lower limb pain had disappeared; she was advised to continue wearing the elastic stocking. Repeated venous duplex scan showed the same result as the initial assessment. The D-dimer level was reduced to 0.53 mcg/mL FEU (the normal range is 0.22–0.45) in March 2021.

She booked an appointment with family medicine clinics because she was concerned about the continuation of combined oral contraception, i.e., desogestrel/ethinyl oestradiol tablets, with the high D-dimer level and risk of thrombosis. The follow-up D-dimer level in May 2021 was normal (0.4 mcg/mL FEU). Although she was counselled about other types of contraception, especially with respect to her varicose veins, she decided to continue the same contraceptive.

## Discussion and conclusions

The literature shows that D-dimer is commonly elevated in patients with COVID-19 [1–3]. Indeed, D-dimer levels correlate with disease severity and are a reliable prognostic marker for in-hospital mortality in patients admitted for COVID-19 [6, 7]. In patients with COVID-19 who do not require admission, no routine tests for coagulation markers, such as D-dimer level, are required in guidelines [8]. However, 30.1% of patients discharged from three large hospitals in London had persistently elevated d-dimer [9], and the value decreases over time post-COVID [9].

The literature reported a three- to fivefold increased risk of venous thromboembolism (VTE) in users of combined oral contraceptives compared to non-users [10]. Ethinyl oestradiol can increase levels of some coagulation factors and fibrinogen [11, 12]. Additionally, it can decrease plasma levels of anticoagulant factors, including antithrombin and tissue factor pathway inhibitors [11, 12].

The harm depends on the type of progestogen used and the dose of ethinyl oestradiol [4, 5, 12]. Although systematic reviews have suggested that oral progestin-only (POC) contraceptive methods do not increase the risk of venous thromboembolism, injectable POC use might increase it [13, 14].

Another knowledge gap is highlighted as a systematic review concerning OCP users with superficial venous disease found a limited number of studies and quality, i.e. no definitive conclusion can be made about increased VTE risk [15].

According to a panel of Spanish experts from various scientific societies, during reduced mobility of home isolation for mild COVID-19 women, it is recommended to withdraw combined hormonal contraception [16]. If contraception is required, they recommend that combined hormonal contraception can be continued or replaced by progestin-only contraception if there is another risk factor [16]. Interestingly, some patients with mild symptoms of COVID-19 may develop venous thromboembolic events, which indicate a hypercoagulable state [17, 18].

Another group of experts considers it appropriate to discontinue any combined hormonal contraceptives in patients with suspected or confirmed COVID-19 [19]. As the risk of venous thromboembolism is lower with OCP containing second-generation progestins than OCP containing third-generation progestins, they recommend using OCP containing second-generation progestins such as norgestrel or levonorgestrel [19–21]. A Cochrane review is carried out to determine whether the use of hormonal contraception increases the risk of venous and arterial thromboembolism in women with COVID-19 [5].

COVID-19 and OCP can both cause thromboembolic manifestations. Studies should evaluate the risk of venous thromboembolism among post-COVID-19 women who are on combined oral contraceptives. The current OCP guidelines for the general population should be applied for post-COVID-19 users until further scientific evidence is available.

## Data Availability

Data sharing is not applicable to this article as no datasets were generated or analyzed during the current study.

## Abbreviations

COVID-19: Coronavirus disease 2019
DVT: Deep vein thrombosis
OCP: Oral combined contraception
VTE: Venous thromboembolism
POC: Progestin-only contraception
